# HIV incidence among people who inject drugs in the Middle East and North Africa: mathematical modelling analysis

**DOI:** 10.1002/jia2.25102

**Published:** 2018-03-25

**Authors:** Ghina R Mumtaz, Susanne F Awad, Ali Feizzadeh, Helen A Weiss, Laith J Abu‐Raddad

**Affiliations:** ^1^ Infectious Disease Epidemiology Group Weill Cornell Medicine ‐ Qatar Cornell University Doha Qatar; ^2^ Department of Infectious Disease Epidemiology Faculty of Epidemiology and Population Health London School of Hygiene and Tropical Medicine London UK; ^3^ Regional Support Team for the Middle East and North Africa Joint United Nations Programme on HIV/AIDS Cairo Egypt; ^4^ MRC Tropical Epidemiology Group Department of Infectious Disease Epidemiology Faculty of Epidemiology and Population Health London School of Hygiene and Tropical Medicine London UK; ^5^ Department of Healthcare Policy and Research Weill Cornell Medicine Cornell University NY USA; ^6^ College of Public Health Hamad bin Khalifa University Doha Qatar

**Keywords:** HIV, people who inject drugs, Middle East and North Africa, incidence, prevalence, mathematical modelling, intervention

## Abstract

**Introduction:**

Emerging HIV epidemics have been documented among people who inject drugs (PWID) in the Middle East and North Africa (MENA). This study estimates the HIV incidence among PWID due to sharing needles/syringes in MENA. It also delineates injecting drug use role as a driver of the epidemic in the population, and estimates impact of interventions.

**Methods:**

A mathematical model of HIV transmission among PWID was applied in seven MENA countries with sufficient and recent epidemiological data and HIV prevalence ≥1% among PWID. Estimations of incident and/or prevalent infections among PWID, ex‐PWID and sexual partners of infected current and ex‐PWID were conducted.

**Results:**

The estimated HIV incidence rate for 2017 among PWID ranged between 0.7% per person‐year (ppy) in Tunisia and 7.8% ppy in Pakistan, with Libya being an outlier (24.8% ppy). The estimated number of annual new infections was lowest in Tunisia (n = 79) and Morocco (n = 99), and highest in Iran and Pakistan (approximately n = 6700 each). In addition, 20 to 2208 and 5 to 837 new annual infections were estimated across the different countries among sexual partners of PWID and ex‐PWID respectively. Since epidemic emergence, the number of total ever acquired incident infections across countries was 706 to 90,015 among PWID, 99 to 18,244 among sexual partners of PWID, and 16 to 4360 among sexual partners of ex‐PWID. The estimated number of prevalent infections across countries was 341 to 23,279 among PWID, 119 to 16,540 among ex‐PWID, 67 to 10,752 among sexual partners of PWID, and 12 to 2863 among sexual partners of ex‐PWID. Increasing antiretroviral therapy (ART) coverage to the global target of 81% – factoring in ART adherence and current coverage – would avert about half of new infections among PWID and their sexual partners. Combining ART with harm reduction could avert over 90% and 70% of new infections among PWID and their sexual partners respectively.

**Conclusions:**

There is considerable HIV incidence among PWID in MENA. Of all new infections ultimately due to injecting drug use, about 75% are among PWID and the rest among sexual partners. Of all prevalent infections ultimately attributed to injecting drug use as epidemic driver, about half are among PWID, 30% among ex‐PWID and 20% among sexual partners of PWID and ex‐PWID. These findings call for scale‐up of services for PWID, including harm reduction as well as testing and treatment services.

## 1 Introduction

As part of the global commitment to end the AIDS epidemic by 2030, the Joint United Nations Programme on HIV/AIDS (UNAIDS) stipulated the ambitious fast‐tracking agenda including a “90‐90‐90” target calling to diagnose 90% of people living with HIV/AIDS (PLHIV), provide antiretroviral therapy (ART) for 90% of those diagnosed, and achieve viral suppression for 90% of those treated 1. Also, on the fast track agenda is to reduce the number of new infections to below 500,000 by 2020 2. With gaps persisting along this HIV cascade, the UN General Assembly agreed in 2016 that staying on the Fast‐Track to ending AIDS by 2030 would only be possible if key populations (KPs) at higher risk of infection have access to comprehensive prevention services 3.

The Middle East and North Africa (MENA), which we define to include 24 countries from Morocco in the West to Afghanistan and Pakistan in the East, is one of few regions where the number of new HIV infections is increasing 3. The vast majority of these infections seem to be occurring among KPs, including people who inject drugs (PWID) and their sexual partners 3. Emerging HIV epidemics have been recently documented among PWID in one‐third of MENA countries, with a risk environment suggesting potential for further HIV spread 4, 5. As a central population to the epidemic in several countries 4, PWID are a priority population if the Fast‐Track targets are to be achieved in this region 2.

PWID remain, globally and overall more so in MENA, one of the hardest‐to‐reach and most stigmatized KPs, which impedes the collection of epidemiological data (both risk group size estimates and biological measures) to track PWID through the HIV cascade and inform policy 2, 6, 7, 8. Despite noticeable progress in the collection of HIV prevalence data in MENA, HIV incidence data remain scarce among all populations groups 4. Among PWID, only three cohort‐type HIV incidence studies have been conducted; in Afghanistan 9, Pakistan 10 and Iran 11, and all indicated substantial incidence 4. Quantifying HIV incidence among PWID in MENA is urgently needed to provide baseline data to track progress towards UNAIDS target of reducing the number of new HIV infections among young people and adults by 75% by 2020 2.

In this study, we use mathematical modelling to estimate, at country‐level in MENA, HIV incidence among PWID due to non‐sterile drug injections. We also estimate HIV incidence among their sexual partners due to heterosexual sex with infected PWID, and delineate the role of injecting drug use as a driver (that is the factor behind direct and onward transmission) of the HIV epidemic in the population. Our approach is comprehensive and aims to explore and quantify the different HIV transmission pathways in the population that are initiated by injecting drug use.

Specific objectives are to estimate: (1) the number of incident HIV infections among PWID and their sexual partners, (2) the total number of HIV infections that were acquired among PWID and their sexual partners since the emergence of the PWID HIV epidemic, (3) the number of HIV infections among ex‐PWID and their sexual partners, where ex‐PWID are individuals who acquired HIV infection while injecting but have stopped injecting in the past year, and hence could be missed by programmes targeting current drug users, and (4) the impact of select interventions on HIV incidence among PWID and their sexual partners.

## 2 Methods

This study included seven MENA countries with sufficient epidemiological data and a current HIV prevalence ≥1% among PWID 4. These are Afghanistan, Egypt, Iran, Libya, Morocco, Pakistan and Tunisia. The remaining 16 MENA countries have either zero or unknown HIV prevalence among PWID 4, and hence estimations of incidence were not possible in these countries.

### 2.1 Description of the model

We adapted the Kwon *et al*. mathematical model of parenteral HIV transmission among PWID to estimate HIV incidence 12. This is a cohort‐type model that assumes that sharing of needles/syringes occurs in groups of specific size, where PWID share in random order, and where each PWID injects once per sharing event. HIV transmissions through sharing needles/syringes can then occur in groups containing an infected person.

The model uses input data on HIV prevalence, number of times a needle/syringe is reused, and levels of effective syringe cleaning to estimate number of HIV transmissions per sharing event. The model then estimates HIV incidence in the total PWID population using data on size of the PWID population, frequency of injecting, and levels of sharing. We extended the model by adjusting for the effect of ART (ART efficacy and country‐specific coverage levels in Tables 1 and 2 respectively) and allowing heterogeneity in injecting risk behaviour. We also allowed the number of times a needle/syringe is reused to be a function of the sharing group size (model structure and equations in Additional file 1).

**Table 1:** Model assumptions in terms of parameter values

Parameter	Value	Source
Biological parameters		
Transmission probability per unsterile injection	0.007	Systematic review and meta‐analysis 39 and long‐term cohort study 40
Transmission probability per unprotected coital act (non‐commercial)	0.003	Systematic review and meta‐analysis 41
Efficacy of ART in reducing HIV transmission	0.96	Clinical trial of treatment for prevention and other observational data 42, 43
Effectiveness of ART in reducing HIV transmission	0.69	Calculated as the product of ART efficacy and adherence
Epidemiology parameters		
Total number of PWID	See Table 2	MENA PWID data 4, 44, 45, 46, 47, 48
ART coverage	See Table 2	UNAIDS country estimates for ART coverage among all people living with HIV/AIDS in 2015 49
HIV prevalence among PWID	See Table 2	MENA PWID data 4
HIV prevalence among sexual partners	One‐third of HIV prevalence in PWID	Bio‐behavioural survey in Iran 50, consistent with similar modelling work in the region 29
Natural history parameters		
Natural mortality rate per year	0.020	Cohort studies 51, 52
HIV disease mortality rate per year	0.091	UNAIDS data compilation 53 and cohort studies 54, 55, 56
Behavioural parameters		
Adherence of PWID to ART	0.72	Systematic review and meta‐analysis 57
Stopping injection rate per year	Lib: 0.04 All countries: 0.10	MENA PWID data 4
Number of years of injecting after seroconversion	Lib: 6.8 years All countries: 4.7 years	Estimated from the natural mortality rate, HIV disease mortality rate and stopping injection rate
Average size of sharing group (# of sharing partners)	Ira: 3, Pak: 2, Others: 3	Iran and Pakistan: model fitting to epidemiological data Others: informed by Iran and Pakistan fitted values and epidemiological data from Iran 22, 23
Proportion of PWID who inject daily	0.50	Global survey data 12, 58
Number of injections per PWID per day among those who inject daily	Ira: 3.3, Pak: 2.2, Others: 2.2	Iran and Pakistan: country‐specific epidemiological data 4 Others: median of all MENA measures 4
Average time between two subsequent injections for PWID who inject less frequently than daily	14 days	Global survey data 12
Average frequency of injecting per PWID per year	Ira: 602, Pak: 402, Others: 402	Calculated as a weighted average of daily and non‐daily injectors 12
Proportion of PWID who share injections	Afg: 0.29, Egy: 0.40, Ira: 0.31, Lib: 0.45, Mor: 0.35, Pak: 0.60, Tun: 0.30	MENA PWID data 4
Proportion of the injections that are shared for PWID who share injections	Egy: 0.62, Ira: 0.28, Mor: 0.52, Pak: 0.40, others: 0.40	Egypt, Iran and Pakistan: country‐specific epidemiological data 4 Others: calculated using other MENA measures 4
Average number of times a shared needle/syringe is used before disposal	Equal to the size of the sharing group, with a maximum value of 10	
Proportion of PWID with regular sexual partners in the last year	0.660	MENA PWID data 4
Proportion of PWID with non‐regular sexual partners in the last year	0.337	MENA PWID data 4
Number of yearly coital acts with regular sexual partners	50	Bio‐behavioral survey in Iran 50
Number of yearly coital acts with non‐regular sexual partners	20	Bio‐behavioral survey in Iran 50
Condom use with regular sexual partners in the last act	0.295	MENA PWID data 4
Condom use with non‐regular sexual partners in the last act	0.359	MENA PWID data 4
Needle/syringe cleaning parameters		
Effectiveness of needle/syringe cleaning	0.75	Modelling work 12 based on 59 and 60
Proportion of shared injections that are cleaned	0.15	MENA PWID data 4, & modelling work 61 based on 62

Afg, Afghanistan; ART, antiretroviral therapy; Egy, Egypt; Ira, Iran; Lib, Libya; MENA, Middle East and North Africa; Mor, Morocco; Pak, Pakistan; PWID, people who inject drugs; Tun, Tunisia.

**Table 2:** HIV infection estimations among people who inject drugs and their heterosexual sex partners in the Middle East and North Africa

Country		Afghanistan	Egypt	Iran	Libya	Morocco	Pakistan	Tunisia
HIV PWID epidemic characteristics								
Total number of PWID 4, 44, 45, 46, 47, 48	n	18,820	90,809	1,85,000	4446	3000	117,632	11,000
Date of HIV epidemic emergence among PWID 4	Year	2009	2008	2001	2000	2008	2004	2009
HIV epidemic state among PWID 4	Level‐trend	Concentrated ‐ Emerging	Concentrated ‐ Emerging	Concentrated ‐ Established	Concentrated‐Unknown	Concentrated ‐ Emerging	Concentrated ‐ Emerging	Low‐level
Most recent representative HIV prevalence 4 (national and/or population‐adjusted)	% (95% CI)	4.4 (3.3 to 5.7)	7.2 (5.2 to 9.6)	15.1 (13.7 to 16.6)	87.1 (81.5 to 91.9)	11.5 (9 to 14.5)	27.2 (26.0 to 28.5)	3.1 (3.1 to 1.9)
HIV incidence in the total population 16	n (range)	1000 (500 to 2700)	1500 (1000 to 2800)	7100 (4400 to 16,000)	No data	1200 (1000 to 1600)	17,000 (12,000 to 30,000)	500 (200 to 500)
Number of PLHIV 16	n	6900	11,000	73,000	No data	24,000	100,000	2600
ART coverage among PLHIV 49	%	5.3	18.7	8.7	16.7^a^	36.7	6.1	28.3
Current year estimations of:								
HIV incidence rate in PWID	% ppy (95% UI)^c^	1.2 (0.8 to 2.4)	3.8 (2.1 to 6.6)	4.4 (2.3 to 7.1)	24.8 (13.3 to 41.3)	3.7 (2.0 to 6.3)	7.8 (4.3 to 13.4)	0.7 (0.4 to 1.4)
HIV incidence in PWID	n (95% UI)^c^	214 (124 to 446)	3217 (1639 to 5847)	6773 (3328 to 11,842)	142 (73 to 262)	99 (49 to 175)	6679 (3369 to 11,997)	79 (41 to 152)
Contribution of PWID to total incidence	% (95% UI)^c^	21.4 (12.4 to 44.6)	NA	95.4 (46.9 to 100)	No data	8.2 (4.1 to 14.6)	39.3 (19.8 to 70.6)	15.9 (8.2 to 30.4)
HIV incidence in sexual partners of infected current PWID	n (95% UI)^c^	62 (33 to 109)	442 (226 to 787)	1977 (1112 to 3218)	193 (109 to 311)	20 (11 to 35)	2208 (1239 to 3566)	22 (10 to 42)
HIV incidence in sexual partners of infected ex to PWID	n (95% UI)^c^	15 (9 to 26)	87 (49 to 148)	720 (441 to 1095)	–b	5 (3 to 8)	837 (521 to 1261)	6 (3 to 11)
Estimated total number of incident HIV infections, since epidemic emergence, among:								
PWID	n	1753	12,257	82,069	–b	706	90,015	764
Sexual partners of infected current PWID	n	331	1892	17,369	–b	99	18,244	125
Sexual partners of infected ex‐PWID	n	54	301	4360	–b	16	4338	21
Estimated total number of prevalent HIV infections among:								
Current PWID	n	829	6553	28,139	–b	347	32,279	341
Ex‐PWID	n	303	1875	14,484	–b	119	16,540	136
Sexual partners of infected current PWID	n	224	1293	9640	–b	67	10,752	84
Sexual partners of infected ex‐PWID	n	40	219	2692	–b	12	2863	16

CI, confidence interval; PLHIV, people living with HIV/AIDS; PWID, people who inject drugs; ppy; per person‐year; UI, Uncertainty interval.

a No data were available – the median ART coverage across all MENA countries was used.

b The measured HIV prevalence in Libya was not consistent with reported levels of risk behaviour; hence estimations of past exposures were not possible.

c Result of the uncertainty analysis varying model parameters by 25%.

To account for heterogeneity in risk behaviour, we assumed that the size of the sharing group in each country follows a gamma distribution. This is a widely used probability distribution to model heterogeneity in diverse biological, ecological and physical phenomena. Since the gamma distribution is right skewed, it assumes that the majority of the PWID population share injections in smaller groups whereas a small fraction shares in larger groups (such as at shooting galleries), as suggested by behavioural and qualitative studies 4, 8. In the absence of clear data to parameterize this variability in injecting risk behaviour, the parameters of the gamma distribution were informed by available data on the variability in sexual risk behaviour and networking, and assumed that the variance of the gamma distribution is equal to its mean 13.

We extended the model to include onward transmission to sexual partners of PWID whereby infected PWID may transmit the infection to their heterosexual sex sexual partners through unprotected sex. The number of incident infections among sexual partners of PWID was estimated using input data on the proportion of PWID who had a sexual partner in the last year, HIV prevalence among PWID and among their sexual partners, and the annual number of unprotected coital acts per partnership (Additional file 1).

### 2.2 Data sources

The model parameters were based on recent empirical HIV natural history and epidemiology data (Table 1). Whenever available, country‐specific parameter values were used, as informed mainly by a recent systematic review of HIV and PWID in MENA 4. One example is HIV prevalence data where we used, for each MENA country, the most recent representative HIV prevalence among PWID as identified through the review 4. Data from integrated bio‐behavioural surveillance surveys were preferred over prevalence data generated through other convenience cross‐sectional studies. National‐level estimates from these studies, when available, were used. In countries with specific location (such as city‐based) prevalence measures, the mean HIV prevalence was calculated across these measures. In four out of the seven countries included in our study (Afghanistan, Iran, Pakistan and Tunisia), HIV prevalence data came from multiple cities/provinces, and in two of these countries (Iran and Pakistan), the studies had a national coverage with 10 to 16 cities/provinces included 4 (Table 2). Libya is the only country where a high quality integrated bio‐behavioiral survey was conducted in only one city, Tripoli, whereas two cities contributed to the prevalence in Egypt and Morocco 4 (Table 2).

Country‐specific parameter values were complemented by global and MENA‐wide aggregate data 4, as needed (Table 1). Model fitting was used to derive one parameter, the average size of the sharing group (mean of the gamma distribution). We used a deterministic compartmental model 14 to fit the trend in HIV prevalence in two countries with sufficient trend data (Iran and Pakistan) 4, and then used the estimated incidence rate and the adapted Kwon *et al*. model to fit the value of the sharing group size (Table 1). Fitting was implemented by minimizing the residual sum of squares between all data points and model predictions 15.

### 2.3 Plan of analysis

**Figure 1:**
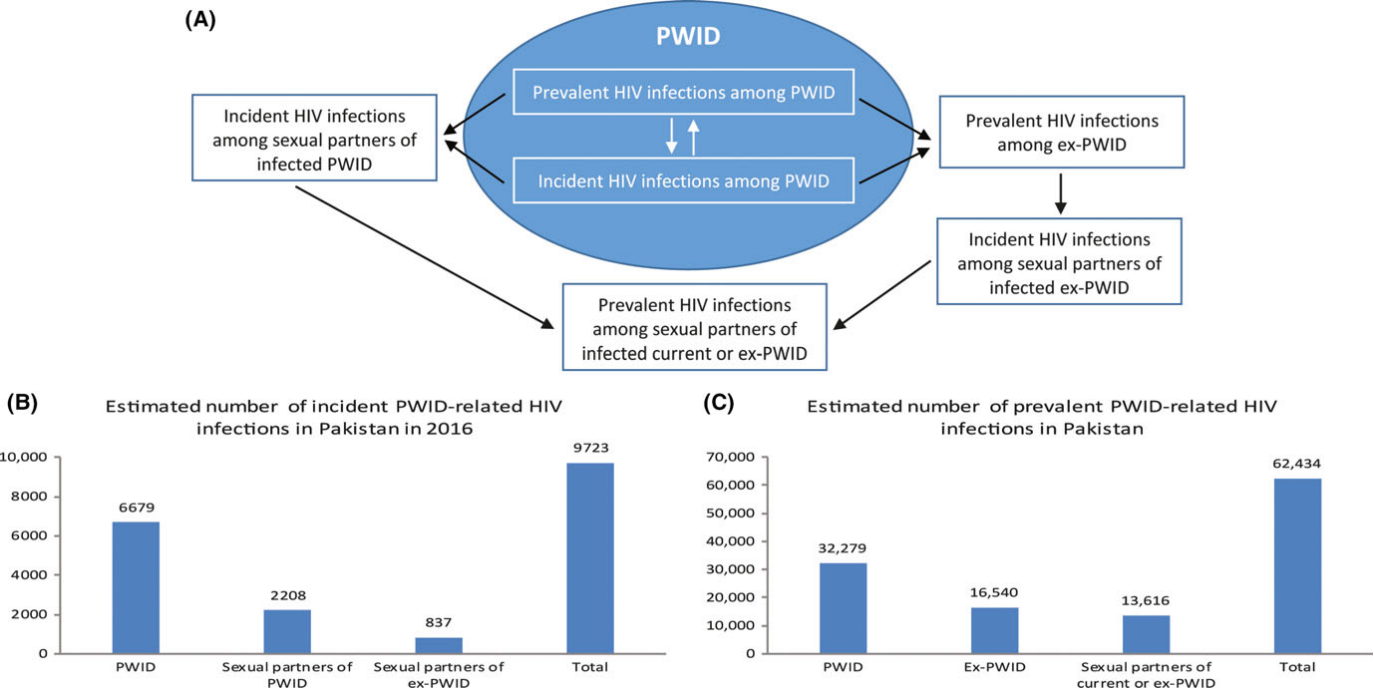
HIV transmission pathways in the population arising from injecting drug use. Panel **(A)** displays the various HIV transmission pathways that are due to injecting drug use, starting among people who inject drugs (PWID) and percolation of infection to the wider non‐injecting community – mainly sexual partners of current and ex‐PWID. The blue oval refers to the PWID population, while the white space around it refers to the general population. Black arrows refer to transmission chains. Panels **(B)** and **(C)** provide one example from Pakistan for the number of incident **(B)** and prevalent **(C)** infections that are caused by these transmission pathways and that affect the different members of the injecting and non‐injecting communities.

We conducted, in each of the seven countries included, the following estimations that capture the different HIV transmission pathways arising from injecting drug use in the population (Figure 1A). They include estimations of incident and/or prevalent infections among PWID, ex‐PWID, and sexual partners of infected current and ex‐PWID. We estimated incidence due to heterosexual sex between infected PWID and their partners. HIV incidence was defined as the number of new infections per year, and incidence *rate* the number of new infections per susceptible person per year.

#### 2.3.1 Current year estimations

The model was applied at country‐level to estimate HIV incidence and rate among PWID for the current year. Estimated incidence among PWID was compared to the number of incident HIV infections in the total population as estimated by UNAIDS Spectrum model 16. The number of incident infections among sexual partners of PWID and of ex‐PWID living with HIV for the current year was estimated. Separate parameterization and estimations were made for regular and non‐regular sexual partners, with their sum reported in the results.

#### 2.3.2 Analysis of past infections

We estimated the number of HIV infections that have ever been acquired among PWID in each country (past infections; that is infections that occurred before the current year) by retracing the course of the HIV epidemic among PWID starting from the year of epidemic emergence, informed by epidemiological data 4 (Table 2). We started with 1% HIV prevalence among PWID at the start of the epidemic HIV transmission and, in each country, ran the model the number of times equal to years since HIV epidemic emergence. HIV prevalence in each year was recalculated by adding the number of incident infections in this year to the number of prevalent infections from previous years, while adjusting for PWID who (a) stopped injecting, or (b) died from “natural” mortality (all causes, including overdose), or (c) died from HIV disease mortality.

Iterating this process over time provides an estimate for HIV prevalence in the last year. As the observed HIV prevalence tended to be higher than the estimated prevalence in the last year, we increased the level of risk behaviour in the first iteration, accounting for higher risk behaviour in earlier years of the epidemic, and used linear interpolation to reach observed HIV prevalence levels at the last year. A sensitivity analysis was conducted in one country, Iran, assuming an exponential, rather than linear, decrease in injecting risk behaviour over time. In Libya, the measured HIV prevalence (87% 17) was not consistent with reported levels of current risk behaviour and hence, estimations of past exposures were not possible.

The past infections model was also used to estimate the number of incident cases that ever happened among sexual partners of infected current and ex‐PWID since HIV epidemic emergence. The prevalent number of infected current/ex‐PWID, and their partners were estimated.

#### 2.3.3 Uncertainty analysis

Multivariate uncertainty analysis was conducted to specify the range of uncertainty in the estimated HIV incidence for the current year among PWID and their sexual partners. Two sets of uncertainty analyses were conducted, one where model parameters were varied within 25% of their point estimates, as a reasonable variation based on existing similar modelling studies 18, 19, and another more conservative analysis with 50% variation around model parameters values. In both, HIV prevalence was varied within its measured 95% confidence interval (Table 2). We implemented 10,000 runs using Monte Carlo sampling from uniform probability distributions for the uncertainty in parameters. 95% uncertainty intervals (UI) for the estimates were determined.

#### 2.3.4 Impact of interventions

We examined the effect of select interventions targeted at PWID: (1) Reducing current sharing of needles/syringes by 25%, 50%, and 75% on HIV incidence among PWID, (2) Introducing opioid substitution therapy (OST) resulting in 10% reduction in the number of PWID and a 10%, 20%, or 30% reduction in the frequency of injecting, on HIV incidence among PWID, (3) Expanding ART coverage among PWID based on most recent test‐and‐treat World Health Organization (WHO) guidelines 20, 21 to reach coverage levels of 25%, 50%, and the global target of 81% 3, on HIV incidence among PWID and their sexual partners, and (4) Increasing current condom use by 25%, 50%, and 75% on HIV incidence among sexual partners. Of note, that the impact of ART is dependent on adherence and existing coverage levels in each country.

We also examined the impact of two packages that include a combination of the above‐mentioned interventions. These packages bracket the realm of plausibility for interventions within the MENA context and have been reached, at least in part, in other regions, such as the impressive scale‐up of ART in resource‐limited settings in sub‐Saharan Africa. The less optimistic scenario includes reducing sharing by 25%, reducing the number of PWID by 10% and the frequency of injecting by 10% (effect of OST), increasing ART coverage to 50%, and increasing condom use by 25%. The more optimistic scenario includes reducing sharing by 75%, reducing the number of PWID by 10% and the frequency of injecting by 30%, increasing ART coverage to 81%, and increasing condom use by 75%.

## 3 Results

The size of the sharing group as estimated through model fitting was two in Pakistan and three in Iran (Table 1). We used a sharing group size of three for the remaining countries, as a rounded‐up average of our estimates in Pakistan and Iran. This sharing group size is in line with both, the global literature 12 and behavioural data from MENA 22, 23.

Results of all main estimations of HIV incidence among PWID and their sexual partners are in Table 2. Table 2 features also results of the uncertainty analysis with 25% variation around model parameters, whereas the impact of a 50% variation on our estimations can be found in Additional File 1, Table 2. The estimated incidence rate among PWID was lowest in Tunisia and Afghanistan at 0.7% (95% UI: 0.4% to 1.4%) and 1.2% (95% UI: 0.8% to 2.4%) per person‐year (ppy) respectively, and highest in Pakistan at 7.8% ppy (95% UI: 4.3% to 13.4%). Libya, at an HIV prevalence of 87% among PWID 17, was an outlier with an estimated incidence rate of 24.8% ppy (95% UI: 13.3% to 41.3%). The incidence rate in remaining countries was around 4% ppy. The estimated number of incident infections for the current year was lowest in Tunisia (n = 79) and Morocco (n = 99), and highest in Iran and Pakistan (around 6700 each). These PWID incident infections represent over 90% of all incident cases in the total population in Iran, 39% in Pakistan, 16% to 21% in Tunisia and Afghanistan, and 8% in Morocco. In addition, 20 to 2208 and 5 to 837 new infections were estimated at country‐level among sexual partners of current and ex‐PWID for this year respectively (Table 2).

In total, we estimated that about 82,000 to 90,000 infections happened among PWID since the start of the PWID HIV epidemic in Iran and Pakistan each; over 12,250 happened in Egypt; 1753 happened in Afghanistan; and over 760 happened in Morocco and Tunisia each (Table 2). Similarly, up to 18,244 and 4360 HIV infections were among sexual partners of current and ex‐PWID respectively, in each of Iran and Pakistan since the start of the PWID HIV epidemic. After accounting for stopping injection and mortality, we estimated that there are currently, at country‐level, 347 to 32,279 prevalent HIV infections among current PWID, 119 to 16,540 among ex‐PWID, and 67 to 10,752 and 12 to 2863 among sexual partners of current and ex‐PWID respectively. The lowest numbers of prevalent infections were in Morocco and Tunisia, while the highest were in Iran and Pakistan (Table 2). Sensitivity analysis indicated a minor impact of the change in the distribution of injecting risk behaviour over the course of the HIV epidemic on these findings (Additional File, Table 3).

**Table 3:** Number and proportion of HIV infections averted due to select interventions in comparison with baseline (current year) estimations of incidence among people who inject drugs and their heterosexual sex partners in the Middle East and North Africa

	Afghanistan	Egypt	Iran	Libya	Morocco	Pakistan	Tunisia
	n (%)	n (%)	n (%)	n (%)	n (%)	n (%)	n (%)
People who inject drugs							
Reducing sharing needles/syringes by 25%	53 (25)	804 (25)	1693 (25)	36 (25)	25 (25)	1670 (25)	20 (25)
Reducing sharing needles/syringes by 50%	107 (50)	1608 (50)	3386 (50)	71 (50)	49 (50)	3340 (50)	40 (50)
Reducing sharing needles/syringes by 75%	160 (75)	2412 (75)	5080 (75)	107 (75)	74 (75)	5009 (75)	60 (75)
Impact of OST:							
Reducing the number of PWID by 10% and:							
reducing injecting frequency by 10%	40 (19)	611 (19)	1287 (19)	27 (19)	19 (19)	1269 (19)	15 (19)
reducing injecting frequency by 20%	60 (28)	901 (28)	1869 (28)	40 (28)	28 (28)	1870 (28)	22 (28)
reducing injecting frequency by 30%	79 (37)	1190 (37)	2506 (37)	53 (37)	37 (37)	2471 (37)	29 (37)
Increasing ART coverage among PWID to 25%	30 (14)	161 (50)	810 (12.0)	9 (6.5)	NA^a^	909 (13.6)	NA^a^
Increasing ART coverage to 50%	68 (32)	798 (24)	2053 (30)	37 (26)	12 (12)	2112 (32)	15 (19)
Increasing ART coverage to 81%	116 (54)	1588 (49)	3595 (53)	71 (50)	40 (41)	3604 (54)	36 (45)
Intervention packages:							
Less optimistic scenario^b^	125 (59)	1747 (54)	3906 (58)	78 (55)	46 (47)	3905 (58)	40 (51)
More optimistic scenario^c^	198 (93)	2960 (92)	6272 (93)	131 (92)	90 (91)	6195 (93)	73 (91)
Sexual partners							
Increasing ART coverage to 25%	8 (14)	21 (5)	228 (12)	12 (6)	NA^a^	289 (13)	NA^a^
Increasing ART coverage to 50%	19 (31)	106 (24)	581 (29)	49 (25)	2 (12)	677 (31)	4 (18)
Increasing ART coverage to 81%	33 (53)	214 (48)	1028 (52)	95 (49)	8 (40)	1167 (53)	10 (44)
Increasing condom use by 25%	7 (11)	47 (11)	210 (11)	21 (11)	2 (11)	234 (11)	2 (11)
Increasing condom use by 50%	13 (21)	94 (21)	421 (21)	41 (21)	4 (21)	470 (21)	5 (21)
Increasing condom use by 75%	20 (32)	142 (32)	635 (32)	62 (32)	6 (32)	709 (32)	7 (32)
Intervention packages:							
Less optimistic scenario^b^	28 (45)	172 (39)	856 (43)	77 (40)	6 (29)	978 (44)	7 (34)
More optimistic scenario^c^	45 (72)	304 (69)	1401 (71)	134 (69)	13 (64)	1576 (71)	14 (66)

ART, antiretroviral therapy; OST, opioid substitution therapy; PWID, people who inject drugs.

The effectiveness of ART in reducing HIV transmission is non‐optimal due to adherence issues among PWID (Table 1).

a Baseline ART coverage is greater than 25%.

b Reducing sharing needles/syringes by 25%, reducing the number of PWID by 10% and reducing injecting frequency by 10%, increasing ART coverage to 50%, and increasing condom use by 25%.

c Reducing sharing needles/syringes by 75%, reducing the number of PWID by 10% and reducing injecting frequency by 30%, increasing ART coverage to 81%, and increasing condom use by 75%.

**Figure 2:**
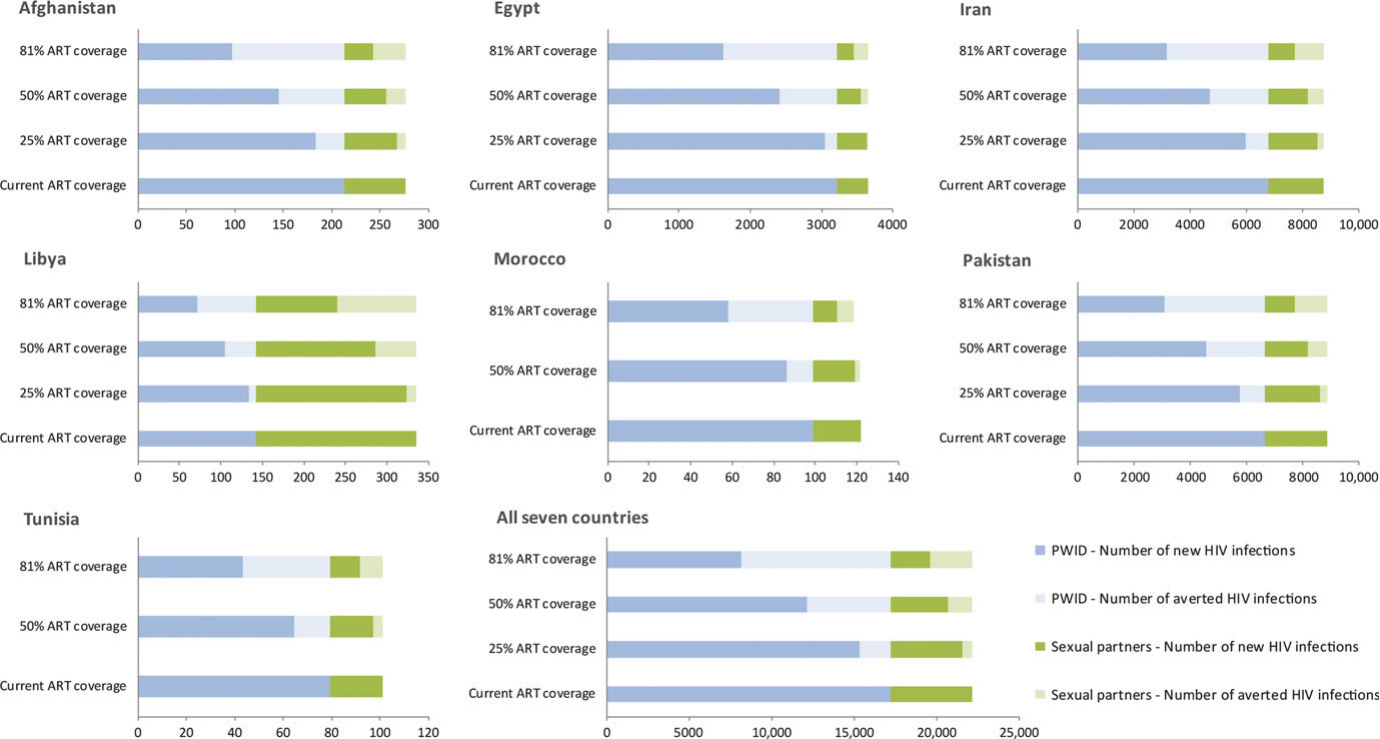
Effect of expanding antiretroviral therapy coverage (ART) on HIV incidence among people who inject drugs (PWID) and their heterosexual sex partners in the Middle East and North Africa (MENA). The graphs display, at various ART coverage levels, the number of new HIV infections and the number of infections averted in comparison with baseline (current year) estimations of HIV incidence among PWID and their heterosexual sex partners.

**Figure 3:**
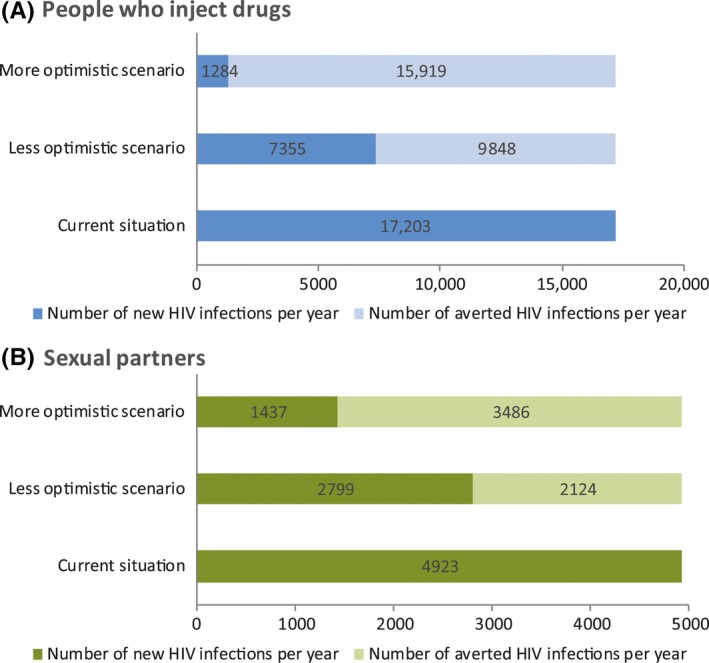
Effect of two comprehensive intervention packages on HIV incidence among people who inject drugs (PWID) **(A)** and their heterosexual sex partners **(B)** in all seven Middle East and North Africa (MENA) countries. The package with the less optimistic scenario includes reducing sharing by 25%, reducing the number of PWID by 10% and reducing injecting frequency by 10% (effect of opioid substitution therapy), increasing ART coverage to 50%, and increasing condom use by 25%. The package with the more optimistic scenario includes reducing sharing by 75%, reducing the number of PWID by 10% and reducing injecting frequency by 30%, increasing ART coverage to 81%, and increasing condom use by 75%. The graphs display the number of new HIV infections and the number of infections averted in comparison with baseline (current year) estimations of HIV incidence among PWID and their heterosexual sex partners.

The impact of interventions on HIV incidence among PWID and/or their sexual partners is in Table 3 and Figures 2 and 3. Reducing sharing by 25%, 50% and 75% respectively was associated with a similar proportional reduction in the number of incident infections among PWID. Reducing sharing by 25% would avert 20 to 53 infections per year in Afghanistan, Libya, Morocco and Tunisia; over 800 infections in Egypt; and about 1700 infections in Iran and Pakistan each. A reduction in sharing by 75% would avert over 5000 infections per year in each of Iran and Pakistan (Table 3).

Introducing OST programmes with various coverage levels was found to lead to 19% to 37% reduction in the number of incident infections among PWID. A reduction in injecting frequency by 30% and in the number of PWID by 10%, as a result of OST, would avert up to 80 infections per year in Afghanistan, Libya, Morocco and Tunisia; over 1100 infections in Egypt; and about 2500 infections in each of Iran and Pakistan (Table 3).

Increasing ART coverage to 25%, 50% and 81% of all infected PWID would reduce incidence among PWID and their sexual partners at country‐level by 5% to 14%, 12% to 32% and 40% to 54% respectively (Table 3, Figure 2). In all seven countries combined, 50% ART coverage would result in 5100 and 1400 infections averted per year among PWID and their sexual partners respectively (Figure 2). Increasing condom use by 25%, 50% and 75% would result in 11%, 21% and 32% reduction in the number of annual infections among sexual partners of PWID (Table 3).

Finally, implementing the less optimistic intervention package would avert 57% (n = 40 to 3906 at country‐level) and 43% (n = 6 to 978) of incident infections per year among PWID and their sexual partners respectively, while the more optimistic scenario would avert 93% (n = 73 to 6272) and 71% (n = 13 to 1576) of these infections respectively (Table 3, Figure 3).

## 4 Discussion

A relatively high HIV incidence rate among PWID was found in most MENA countries with at least 1% prevalence among PWID (range: 4% to 8% ppy), supporting recent analyses indicating concentrated and at times rapidly growing HIV epidemics among PWID 4. A lower incidence rate of about 1% ppy was estimated in Afghanistan, where epidemiological data points to a nascent localized HIV epidemic among PWID 4, and in Tunisia where the PWID HIV epidemic appears to be at low level 4. Libya stood out with an estimated incidence rate of 25% ppy. This reflects the very high 87% HIV prevalence among PWID found in the best study in this country – one of the highest prevalence ever reported among PWID globally, a figure difficult to interpret and generalize for all of Libya in view of the limited data in this country 4, 17.

Our estimates of HIV incidence among PWID are overall consistent with our epidemiological understanding of the HIV epidemic among PWID in each country 4, and are congruent with the estimated size of the epidemic in the whole population per UNAIDS Spectrum estimates 16. For example, PWID contributed the vast majority of HIV infections in Iran, a country with an established HIV epidemic among PWID 4, 5, 14 and where injecting drug use is the main driver of the epidemic at the national level 4, 24. Similarly, a small contribution of PWID to total incidence was found in Tunisia and Morocco; two countries where the HIV epidemic is mainly focused among men who have sex with men (MSM) and commercial sex networks respectively 25, 26, 27, 28, 29.

We also found that a substantial number of HIV infections in the general population are linked to infections among PWID, due to individuals who acquired the infection in the past through drug injection, but are no longer injecting, and due to onward transmission to sexual partners. We estimated that about 30% of incident HIV infections that are due to injecting drug use are among sexual partners of current/ex‐PWID, and about half of prevalent infections are among ex‐PWID and sexual partners of current/ex‐PWID (Figure 1B and C). Figure 1 highlights how injecting drug use drives HIV transmission not only among current PWID but also among individuals with no or no recent injecting drug use. Our findings agree with recent Mode of Transmission analyses in the region that estimated a substantial number of HIV infections among sexual partners/spouses of persons engaging in HIV high‐risk behaviour such as PWID, clients of female sex workers and MSM 24, 27, 29.

Despite the growing epidemics among PWID, the HIV response remains limited in MENA. By 2014, needle and syringe exchange programmes (NSP) were available in nine countries, and OST in five 30. MENA has the lowest ART coverage globally at a median of 17% in 2015 31, and could not reach the 2015 mid‐term regional objective of 50% coverage under WHO's initiative to end MENA's HIV treatment crisis 32. The treatment cascade among PWID seems to suggest an even harsher reality 33. The striking gap between regional figures and the 90‐90‐90 target is of great concern, especially since the epidemic in this region is strongly driven by injecting drug use. The punitive legal environment, stigma around HIV testing, and fear of discrimination are all challenges that hamper uptake of treatment by PWID 3. These challenges need to be addressed to scale‐up testing, access to ART, and retention in care. We estimated that increasing ART coverage to the global target of 81% (90% of 90%), would alone avert close to half of incident infections among PWID and their sexual partners. Combining ART scale‐up with harm reduction – including NSP, OST and condom distribution – as part of an optimistic package would avert over 90% and 70% of infections among PWID and their sexual partners respectively (Figure 3). Furthermore studies and considerations – including a discussion dialogue with programme managers, NGOs, affected communities, and other stakeholders – are needed to assess implementation and coverage of these and other interventions, as well as potential barriers and challenges within the MENA context, to reach the desired targets.

There were several limitations in our study. Though we used an elaborate mathematical model structure, our results may depend on the model structure used. For example, we did not account for sexual transmission of HIV among PWID, for anal sex with same‐sex partners, and for further onward transmission beyond the direct spouses/sexual partners. This implies that the impact of PWID as drivers of HIV infection in the population is even larger than what we have estimated with our model. The model also assumes essentially a static structure of injecting groups and does not strictly factor in the full dynamics of partnership formation and dissolution.

Furthermore, estimations were possible in only seven MENA countries with sufficient epidemiological data indicating HIV transmission among PWID. In a number of countries, there are no data about HIV prevalence in PWID, and these countries were naturally not included in our analysis. In others such as Lebanon and Jordan, some studies have been conducted but, in the samples collected, there were no HIV‐positive cases. While there might be hidden HIV epidemics among PWID, or some small background transmission, these were not captured by available studies. This, however, may also truly reflect that HIV has not yet been sufficiently introduced to PWID networks to start epidemic transmission in these countries.

We also assumed that available HIV bio‐behavioural data in each included country is representative of the epidemic at national level and also assumed that current HIV prevalence is the same as most recent representative HIV prevalence. However, these assumptions are only approximately valid. In countries where HIV prevalence data came from only a few geographical localities, our estimates can be biased if these available data are not representative of HIV prevalence among PWID at the national level. For example, in Libya, the HIV prevalence data came from one city, Tripoli 41, and the scale of the epidemic in other parts of Libya is unknown. Similarly, HIV prevalence in each of Cairo and Alexandria was 7%, but no other localities in Egypt were covered by the IBBSS 58. In Morocco, we used average HIV prevalence of two cities where studies were conducted, and estimated an incidence rate of 3.7% ppy. Running our model separately in the two localities generated an incidence of 0.1% ppy in Tanger and 7.4% ppy in Nador, highlighting the diversity of the epidemic within Morocco (data not shown). There is however solid epidemiological evidence that the HIV PWID epidemic in both Iran and Pakistan has reached high levels at the national level 4.

Also, as with all modelling work, the robustness of our findings depends on the quality of input data. While we used the best‐available data in the region, including many quality integrated bio‐behavioural surveillance surveys (IBBSS) 4, the availability and quality of data varied between countries, and there were no MENA‐specific data on some parameters. Noticeable decrease in Global Fund funding for the conduct of IBBSS in the region, as well as rising conflicts with competing national priorities in several countries, have contributed to some of these data gaps. To accommodate for the uncertainty in input parameters, we conducted uncertainty analyses with wide ranges for parameters.

Despite these limitations, this study fills an important gap in estimating HIV infection levels in MENA. HIV‐related estimates in MENA are typically produced through established mathematical models applied globally such as UNAIDS Spectrum 16 and the Global Burden of Disease (GBD) models 34. While these models provide two different approaches for HIV estimations, their focus is on total population‐level estimates 34, 35, which may overlook the dynamics of infection among hidden KPs such as PWID. In this study, we model HIV transmission specifically among PWID, and use quality input data derived from this same population. Such ‘microscopic’ approach could potentially offer more realistic estimations, and may explain some of the inconsistencies observed between our estimates and those of UNAIDS and GBD. One example is the lower than expected contribution of PWID to total incidence we estimated in Afghanistan and Pakistan – two countries where the epidemic is apparently largely driven by injecting drug use 4. In Afghanistan, a similar HIV incidence rate of 1.5% ppy was measured among PWID in a cohort study 9. Under‐reporting of injecting risk behaviour, hidden HIV epidemics among PWID in sites not covered by the IBBSS, and emerging and/or hidden HIV epidemics among other KPs such as MSM in Pakistan, could be potential explanations to the observed lower contribution of PWID to total incidence. Another example is the exceeding number of incident infections we estimated among PWID in Egypt compared to UNAIDS estimated total number of infections in the population, which could be due to the fact that the concentrated HIV epidemic among PWID may be limited to the two cities where the IBBSS studies were conducted. Careful triangulation of multiple sources of evidence is needed to alleviate some of these apparent contradictions, which in truth may reflect incomplete understanding of HIV epidemiology 25.

In addition to providing estimates of incidence, our study provided insights into HIV epidemic dynamics among PWID. Our analyses indicated that current levels of injecting risk behaviour could not explain observed prevalence nor the speed at which these epidemics rose. While it could be partially due to underreporting of risk behaviour, this suggests that the epidemic was initially spreading among PWID sub‐groups with higher levels of risk behaviour. These findings agree with the contextual understanding of these epidemics. For example, the HIV PWID epidemics in Iran and Pakistan started in prisons 36, where they were ignited by limited access to clean needles/syringes and considerable levels of sharing. In Iran, there are reports of syringes being reused 30 to 40 times in prisons 37, with one study measuring a very high HIV incidence rate of 17.2% ppy among incarcerated PWID in a Tehran prison in 2002 11. In Libya, assuming even extreme levels of risk behaviour at epidemic onset could not retrace the course of the epidemic. Since PWID have relatively short injecting careers and high turnover, it is difficult to understand how HIV prevalence has reached such high level. Possibly, this may reflect a very recent HIV sub‐epidemic among the PWID group that was sampled in Tripoli.

## 5 Conclusion

We estimated substantial HIV incidence among PWID in MENA. This is mainly because some of the largest countries, such as Iran and Pakistan, are affected by HIV PWID epidemics with high HIV prevalence. In several countries, PWID were found to contribute dominantly to HIV incidence. Concerted efforts are needed to bypass persistent barriers from governments, society, and health systems to improve service delivery to PWID and their retention throughout the cascade 6. Comprehensive programmes that include ART, NSP, OST, voluntary testing and counselling, and prevention of sexually transmitted infections should be established and extended to include settings of vulnerability such as prisons 38.

We further estimated that for each currently infected PWID there is one other HIV‐infected person in the general population who acquired the infection through PWID dynamics, either as an ex‐PWID or as a sexual partner of current/ex‐PWID (Figure 1). Innovative, context‐sensitive approaches are needed to reach these populations who are usually missed by programmes targeted at PWID, and a large fraction of whom are women, for appropriate interventions and linkage to care.

HIV surveillance among PWID must be expanded in MENA, mainly through the conduct of repeated rounds of IBBSS. Inclusion of sexual partners of PWID should be considered, as well as population size estimations. Generating such epidemiological data will help monitor HIV epidemics, improve quality of input data for estimation studies, guide policy and programmes, and track PWID through the HIV treatment cascade.

## Competing interests

The authors have no competing interests to declare.

## Authors’ contributions

GM contributed to the conception of the study, conceived and generated simulations, conducted analyses and wrote the first draft of the manuscript. SFA contributed to the coding and simulations. HAW contributed to the conception and design of the study. LJA conceived the study and simulations, and contributed to analyses. All authors have read and agreed with the final version of the manuscript.

## Supporting information


**Additional file 1.** Mathematical models’ description.
