# Supplementary material for: HIV incidence among people who inject drugs in the Middle East and North Africa: mathematical modelling analysis
Source: J Int AIDS Soc. 2018 Mar 25;21(3):e25102. doi: 10.1002/jia2.25102 (PMC5867334; doi:10.1002/jia2.25102)
Supplement: Supplementary file 1 — Additional file 1. Mathematical models’ description. [file JIA2-21-e25102-s001.docx]

**Additional file 1**

**Description of mathematical model and other analyses**

1. **Original Kwon et al model**

Below is the derivation of the original model developed by Kwon et al [1], which we used and further adapted for estimating HIV incidence among people who inject drugs (PWID) in the Middle East and North Africa (MENA). The model is a static cohort-type mathematical model of HIV transmission among PWID who share needles/syringes. The model assumes that sharing of needles/syringes occurs in sharing groups of specific average size, where PWID share needles/syringes in a random order, and where each PWID injects once per sharing event. HIV transmission through sharing needles/syringes can then occur in groups containing an infected person.

**Table 1.** Parameters used in the original Kwon et al model [1]

| **Parameter** | **Description** |
| --- | --- |
| $N$ | Total number of PWID |
| $m$ | Average number of PWID in a sharing group |
| $n$ | Average number of injections per PWID per year |
| $s$ | Proportion of PWID who share injections |
| $q_{0}$ | Proportion of the injections that are shared |
| $r$ | Number of PWID infected in the sharing group |
| $p_{0}$ | HIV prevalence among PWID |
| $\beta$ | Probability of transmission from a contaminated needle/syringe |
| $\varepsilon_{c}$ | Effectiveness of needle/syringe cleaning |
| $P_{c}$ | Proportion of shared injections that are cleaned |
| $\delta_{s}$ | Average number of times a shared needle/syringe is used before disposal |

The following series of equations describe the Kwon et al model:

- Total number of shared injections per year:

$$S=Nnsq_{0}$$

- Average number of sharing events per year:

$$SE=\frac{Nnsq_{0}}{m}$$

- Total number of transmissions per year for all PWID (incidence):

$$I=SE*T$$

where $T$ is the average number of transmissions per sharing event.

Deriving $T$, the average number of transmissions per sharing event:

- Probability of having $r$ infected people in a sharing group of size $m$ using binomial theory:

$$\mathrm{P}_{\mathrm{r}/m}=\left(_{r}^{m}\right)p_{0}^{r}(1-p_{0})^{m-r}=\frac{m!}{r!\left(m-r\right)!}p_{0}^{r}(1-p_{0})^{m-r}$$

- Average number of uninfected who will inject *before* the first infected in a sharing event:

$$U_{b}=\frac{m-r}{r+1}$$

- Average number of uninfected who will inject *after* an infected person in a sharing event:

$$U_{a}=m-r-U_{b}=\frac{mr-r^{2}}{r+1}$$

- Average number of needles/syringes used per sharing event:

$$Sy=\frac{m}{\delta_{s}}$$

- Average number of uninfected who will use the *same needle/syringe after* an infected person in a sharing event:

$$U_{s}=\frac{U_{a}}{Sy}=\frac{mr-r^{2}}{r+1}\frac{\delta_{s}}{m}$$

- Probability of transmission per shared injection:

$$\beta_{c}=\beta(1-p_{c})+\beta p_{c}(1-\varepsilon_{c})=\beta(1-p_{c}\varepsilon_{c})$$

- Number of transmissions in the sharing group (per sharing event):

$$T=\sum_{r=1}^{m-1} U_{s}\beta_{c}\mathrm{P}_{\mathrm{r}/m}=\sum_{r=1}^{m-1} \frac{mr-r^{2}}{r+1}\frac{\delta_{s}}{m}*\beta(1-p_{c}\varepsilon_{c})*\left(_{r}^{m}\right)p_{0}^{r}(1-p_{0})^{m-r}$$

$$T=\frac{\delta_{s}\beta(1-p_{c}\varepsilon_{c})}{m}\sum_{r=1}^{m-1} \frac{mr-r^{2}}{r+1}\left(_{r}^{m}\right)p_{0}^{r}(1-p_{0})^{m-r}$$

- Total number of transmissions per year for all PWID (incidence):

$$I=SE*T$$

$$I=\frac{Nnsq\delta_{s}\beta(1-p_{c}\varepsilon_{c})}{m^{2}}\sum_{r=1}^{m-1} \frac{mr-r^{2}}{r+1}\left(_{r}^{m}\right)p_{0}^{r}(1-p_{0})^{m-r}$$

- Incidence rate:

$$\lambda=\frac{I}{N(1-p_{0})}$$

1. **Modifications to the original Kwon et al model**

We further adapted the above model by Kwon et al [1] using the following extensions:

1. **Antiretroviral therapy (ART)**

We adjusted for the use of antiretroviral therapy (ART) on HIV incidence by incorporating ART effect into the transmission probability per shared injection/coital act:

$$\beta'=\beta(1-\Omega ART)$$

Where:

$\beta'$= ART-adjusted transmission probability per unprotected exposure

$\beta$= Original/unadjusted (for ART) transmission probability per unprotected exposure

$\Omega$= Reduction in HIV transmission per exposure due to ART

ART = Proportion of the population on ART (ART coverage)

1. **Heterogeneity in risk behavior**

The original Kwon et al model [1] uses a fixed level of injecting risk behavior for all PWID. To account for heterogeneity in risk behavior, we accommodated different sizes of the sharing group $m$. Specifically, we assumed that $m$ follows a gamma distribution with a mean equal to $m_{average}$. The gamma distribution is right skewed, and therefore assumes that the majority of the PWID population shares injections in smaller groups whereas a small fraction shares in larger groups (such as at shooting galleries). Therefore $m$ is gamma distributed with shape $(k)$ and scale $(\theta)$ parameters:

$$P(m)=\frac{1}{\Gamma(m)\theta^{k}}m^{k-1}e^{-\frac{m}{\theta}}$$

In absence of clear data to parameterize the variability in injecting risk behavior in a given PWID population, the structure of the model was informed by data on the variability in sexual risk behavior and networking, where we assumed that the variance of the gamma distribution is equal to its mean [2]. Accordingly, the scale parameter $\theta=1$ and the shape parameter $k=m_{average}$.

$m_{average}$ was estimated using a deterministic compartmental model [3] to fit the trend in HIV prevalence in two countries with sufficient available trend data (Pakistan and Iran), and then using the estimated incidence rate and the present adapted Kwon et al model to predict using fitting the value of the sharing group size in our model. The fitting was implemented by minimizing the residual sum of squares between all data points and model predictions [4]. Based on the fitting, a value of $m_{average}=2$ was found for Pakistan and of $m_{average}=3$ for Iran.

In the remaining countries, we did not have sufficient data to inform a country-specific estimation of $m_{average}$. Based on the range of 2-3 obtained for Iran and Pakistan, and as informed by epidemiological data from Iran [5, 6], we used a value of $m_{average}=3$ in the remaining countries.

1. **Needle/Syringe reuse**

In the original Kwon et al model [1], the number of times a needle/syringe is reused before disposal ($\delta_{s}$) is fixed and is independent of the size of the sharing group $m$. Since the model also assumes a fixed total number of injections in the population, fixing $\delta_{s}$ will constrain the transmission system after a certain value of $m$ leading to smaller HIV incidence with larger sharing groups. This was a fair assumption in the study setting of Australia where there are effective needle and syringe exchange programs (NSPs) leading to less needle/syringe reuse, where the size of the sharing group is small ($m=2$), and where HIV incidence among PWID is low [1].

In the MENA context of emerging HIV epidemics among PWID [7], high levels of needle/syringe reuse, and weak interventions including NSPs among PWIDs, we have modified the above assumption by relating the number of times a needle/syringe is reused before disposal ($\delta_{s}$) to the size of the sharing group ($m$). We assumed that if $m<10$ , then $\delta_{s}=10$; that is all the PWID in the sharing group will use the same needle/syringe. If $m\geq10$, then $\delta_{s}=m$; that is a needle/syringe is reused for a maximum of ten times in any sharing event.

1. **Additional analyses**
2. **Estimating HIV prevalence at endemic equilibrium**

To estimate HIV prevalence at endemic equilibrium ($P_{e}$) using the estimated HIV incidence rate, the following generic model was used:

$$S$$

Susceptible

$$I$$

Infected

The above deterministic compartmental mathematical model describes the parenteral transmission of HIV through sharing unsterile needles/syringes among PWID. Individuals become PWID, and hence enter into the PWID population, at a rate $b$. $\mu$ is the natural mortality rate, $\gamma$ is the leaving injecting career rate, and $\alpha$ is the HIV/AIDS disease mortality rate. $\lambda$ is the incidence rate (or force of infection) experienced by the susceptible population $S$. The model is expressed with the below system of coupled differential equations:

$$\frac{dS}{dt}=b-\mu S-\gamma S-\lambda S$$

$$\frac{dI}{dt}=\lambda S-\mu I-\gamma I-\alpha I$$

At endemic equilibrium:

$\frac{dI}{dt}=0$, and hence $P_{e}=\frac{\lambda}{\mu+\gamma+\alpha+\lambda}$

1. **Past exposures in PWID**

To estimate the total number of HIV infections that occurred in PWID since the start of the HIV epidemic among PWID, we retraced the course of the HIV epidemic among PWID at country-level starting from epidemic emergence. The year of epidemic emergence was informed by epidemiological data from each country [7]. We ran, in each country, the model $x$ number of times, where $x$ is the number of years since HIV epidemic emergence. We assumed that at year 0, HIV prevalence is 1% ($p_{y_{0}}=0.01$).

The total number of infected PWID in year 1 includes both prevalent infections from the previous year and incident infections in this year, and is given by:

$$Inf_{y_{1}}=Incident_{y_{1}}+prevalent_{y_{0}}$$

$$Inf_{y_{1}}=I_{y_{1}}+Np_{y_{0}}$$

Assuming that the incident infections occur in the middle of the year, the number of HIV infected PWID who will leave the PWID population in year 1 due to leaving injection ($\gamma$), natural mortality ($\mu$), or disease mortality ($\alpha$) is given by:

$$n_{out}^{y_{1}}=(\frac{I_{y1}}{2}+Np_{y_{0}})\times(\mu+\gamma+\alpha)$$

Therefore, HIV prevalence at the end of year 1 will be given by:

$p_{y_{1}}=\frac{Inf_{y_{1}}-n_{out}^{y_{1}}}{N}$ (Note: we assume a fixed total PWID population size ‘$N$’)

The model is then run for year 2 starting with HIV prevalence $p_{y_{1}}$. The same calculations and processes are repeated $x$ times.

It bears notice that the number of HIV infected PWID who will leave the PWID population in year 1 due only to leaving injection is given by:

$$n_{past}^{y_{1}}=(\frac{I_{y1}}{2}+Np_{y_{0}})\times\gamma$$

Accordingly, the number of HIV infected PWID who left the PWID population due to leaving injection in each subsequent year is calculated the same way.

Iterating this process until the last (current) year will provide an estimate for HIV prevalence in the last year. However, in the runs for countries, we were not often able to reach observed HIV prevalence, with the last year estimated prevalence being lower than observed prevalence. We therefore increased the level of risk behavior at year 0, to account for higher risk behavior in earlier years of the epidemic, and used linear interpolation for the level of injecting risk behavior from year 0 to the last year, in order to reach observed levels of risk behavior and HIV prevalence at the last (current) year.

In Libya, the measured HIV prevalence (87% [8]) was not consistent with reported levels of current risk behavior. With such levels of risk behavior, the maximum current HIV prevalence the model could reach was 52%; and hence, estimations of past exposures were not possible in Libya.

1. **HIV incidence in PWID sexual partners**

In a sero-discordant partnership where the PWID is HIV-infected and the sexual partner (opposite sex; only heterosexual transmission was considered) is seronegative, the probability of HIV transmission after one year is given by:

$$z=1-(1-\beta_{s})^{y(1-c)}$$

where:

$\beta_{s}$= HIV transmission probability per unprotected coital act

$y$= Number of coital acts per year

$c$= Proportion of the coital acts that are protected (condom use at last sex)

The number of sero-discordant partnerships is given by:

$$n_{disc}=Nvp_{0}(1-p_{p})$$

where:

$N$= Total number of PWID

$v$= Proportion of PWID who had a sexual partner in the last year

$p_{0}$= HIV prevalence among PWID

$p_{p}$= HIV prevalence among sexual partners of PWID (which was assumed to be equal to one third of HIV prevalence among PWID)

HIV incidence among PWID sexual partners is therefore given by:

$$I_{fsp}=n_{disc}z$$

$$I_{fsp}=Nvp_{0}(1-p_{p})(1-(1-\beta_{s})^{y(1-c)})$$

1. **Past exposures in PWID sexual partners**

HIV incidence in PWID heterosexual sex partners was calculated for each year since start of the HIV PWID epidemic in each MENA country using the year-specific HIV prevalence measures in PWID ($P_{y_{x}}$) as estimated in section C2 above (Past exposures in PWID).

1. **Additional uncertainty analysis**

In addition to varying our model parameters by 25%, the results of which are described in the main text, we have conducted a more conservative uncertainty analysis where we varied model parameters by 50% around their estimated values. The table below displays our main estimates of HIV incidence among PWID and their sexual partners for the current year along with their respective 95% uncertainty intervals.

Table 2. Estimated HIV incidence for the current year among PWID and their heterosexual sex partners in the Middle East and North Africa

| **Country** |  | **Afghanistan** | **Egypt** | **Iran** | **Libya** | **Morocco** | **Pakistan** | **Tunisia** |
| --- | --- | --- | --- | --- | --- | --- | --- | --- |
| **Current year estimations of:** |  |  |  |  |  |  |  |  |
| **HIV incidence rate in PWID** | **% ppy**  **(95% UI)^‡^** | 1.2  (0.3-2.9) | 3.8  (1.0-10.7) | 4.4  (1.1-11.6) | 24.8  (5.8-65.5) | 3.7  (1.0-10.9) | 7.8  (1.9-20.0) | 0.7  (0.2-2.3) |
| **HIV incidence in PWID** | **n**  **(95% UI) ^‡^** | 214  (40-572) | 3,217  (670-9,999) | 6,773  (1,380-19,528) | 142  (22-341) | 99  (21-313) | 6,679  (1,291-18,651) | 79  (18-263) |
| **Contribution of PWID to total incidence** | **%**  **(95% UI) ^‡^** | 21.4  (4.0-57.2) | NA | 95.4  (19.4-100) | No data | 8.2  (1.7-26.1) | 39.3  (7.6-109.7) | 15.9  (3.5-52.6) |
| **HIV incidence in sexual partners of infected current PWID** | **n**  **(95% UI) ^‡^** | 62  (16-159) | 442  (112-1,164) | 1,977  (511-4,798) | 193  (51-464) | 20  (5-51) | 2,208  (581-5,333) | 22  (5-60) |
| **HIV incidence in sexual partners of infected ex-PWID** | **n**  **(95% UI) ^‡^** | 15  (5-35) | 87  (28-196) | 720  (241-1,515) | --^†^ | 5  (2-11) | 837  (282-1,758) | 6  (2-14) |

^†^ The measured HIV prevalence in Libya was not consistent with reported levels of risk behavior; hence estimations of past exposures were not possible

^‡^ Result of the uncertainty analysis varying model parameters by 50%

1. **Sensitivity analysis**

We conducted a sensitivity analysis in Iran, as an illustrative example, to describe the effect of our assumption regarding the trajectory of the epidemic at country level. When retracing the course of the HIV epidemic, and in order to reach current observed HIV prevalence levels, we assumed higher levels of injecting risk behavior at the start of the epidemic and a subsequent linear decrease in these levels to reach observed figures. In the current sensitivity analysis, we assumed an exponential decrease rather than linear decrease in injecting risk behavior over time, and as such explored the impact of these assumptions on our estimated incidence (Table 3).

**Table 3.** Estimated HIV incidence among PWID and their heterosexual sexual partners using two assumptions regarding injecting risk behavior dynamics over the course of the HIV epidemic in Iran

|  | **(A) Linear decrease in risk behavior** | **(B) Exponential decrease in risk behavior** | **Difference**  **(B minus A)** |
| --- | --- | --- | --- |
| **Estimated total number of incident HIV infections, since epidemic emergence, among:** | n | n | n (%) |
| **PWID** | 82,069 | 84,776 | 2,707 (3.3) |
| **Sexual partners of infected current PWID** | 17,369 | 18,170 | 801 (4.6) |
| **Sexual partners of infected ex-PWID** | 4,360 | 4,664 |  |
| **Estimated total number of prevalent HIV infections among:** |  |  |  |
| **Current PWID** | 28,139 | 28,145 | 6 (0.02) |
| **Ex-PWID** | 14,484 | 14,991 | 508 (3.5) |
| **Sexual partners of infected current PWID** | 9,640 | 9,938 | 299 (3.1) |
| **Sexual partners of infected ex-PWID** | 2,692 | 2,857 | 165 (6.1) |

**References**

1. Kwon JA, Iversen J, Maher L, Law MG, Wilson DP. The impact of needle and syringe programs on HIV and HCV transmissions in injecting drug users in Australia: a model-based analysis. J Acquir Immune Defic Syndr. 2009;51(4):462-9. doi: 10.1097/QAI.0b013e3181a2539a. PubMed PMID: 19387355.

2. Omori R, Chemaitelly H, Abu-Raddad LJ. Dynamics of non-cohabiting sex partnering in sub-Saharan Africa: a modelling study with implications for HIV transmission. Sex Transm Infect. 2015. doi: 10.1136/sextrans-2014-051925. PubMed PMID: 25746040.

3. Akbarzadeh V, Mumtaz GR, Awad SF, Awad SF, Abu-Raddad LJ. HCV prevalence can predict HIV epidemic potential among people who inject drugs: Mathematical modeling analysis, Under review. 2016.

4. Lagarias JC, J. A. Reeds, M. H. Wright,and P. E. Wright. Convergence Properties of the Nelder-MeadSimplex Method in Low Dimensions. SIAM Journal of Optimization. 1998;9(1):112-47.

5. Zamani S, Vazirian M, Nassirimanesh B, Razzaghi EM, Ono-Kihara M, Mortazavi Ravari S, et al. Needle and syringe sharing practices among injecting drug users in Tehran: a comparison of two neighborhoods, one with and one without a needle and syringe program. AIDS Behav. 2010;14(4):885-90. Epub 2008/05/17. doi: 10.1007/s10461-008-9404-2. PubMed PMID: 18483849.

6. Zamani S, Radfar R, Nematollahi P, Fadaie R, Meshkati M, Mortazavi S, et al. Prevalence of HIV/HCV/HBV infections and drug-related risk behaviours amongst IDUs recruited through peer-driven sampling in Iran. Int J Drug Policy. 2010;21(6):493-500. Epub 2010/05/21. doi: 10.1016/j.drugpo.2010.04.006. PubMed PMID: 20483578.

7. Mumtaz GR, Weiss HA, Thomas SL, Riome S, Setayesh H, Riedner G, et al. HIV among people who inject drugs in the Middle East and North Africa: Systematic review and data synthesis. PLoS Med. 2014;11(6):e1001663. Epub 2014/06/18. doi: 10.1371/journal.pmed.1001663. PubMed PMID: 24937136; PubMed Central PMCID: PMC4061009.

8. Mirzoyan L, Berendes S, Jeffery C, Thomson J, Ben Othman H, Danon L, et al. New evidence on the HIV epidemic in Libya: why countries must implement prevention programs among people who inject drugs. J Acquir Immune Defic Syndr. 2013;62(5):577-83. Epub 2013/01/23. doi: 10.1097/QAI.0b013e318284714a. PubMed PMID: 23337363.
